# Effects of fermentation time, baking, and storage on ochratoxin A levels in sourdough flat bread

**DOI:** 10.1002/fsn3.4357

**Published:** 2024-07-21

**Authors:** Nazlı Özdemir, Hülya Gül

**Affiliations:** ^1^ Mycotoxin and Residue Unit, Isparta Food Control Laboratory Directorate Republic of Turkey Ministry of Agriculture and Forestry Isparta Turkey; ^2^ Faculty of Engineering and Natural Sciences, Food Engineering Department Süleyman Demirel University Isparta Turkey

**Keywords:** flatbread, HPLC‐FLD, mycotoxin, traditional

## Abstract

Ochratoxin A (OTA), which is one of the most important mycotoxins in terms of human health, can be found in cereal products such as bread, “bazlama” (traditional flatbread), and pita bread, as well as cereals such as wheat, barley, and corn. This study aimed to determine the effect of different fermentation times, baking, and storage for various periods on the presence of OTA in sourdough bazlama. Bazlama flour was contaminated with OTA concentrations of 5 and 10 μg/kg. After two different fermentation times (1.5 and 3 h), baking at 300 ± 5°C, and storage at room temperature (25 ± 2°C) for 0, 5, and 10 days, the change in OTA levels of bazlama samples was determined by the high‐performance liquid chromatography with fluorescence detector (HPLC‐FLD) method. The effect of different storage periods on the presence of OTA is insignificant. Although a general decrease in OTA level has been determined, it has been found that long‐term fermentation (at least 3 h) was more effective, especially in flours with a high concentration (10 μg/kg) of OTA contamination. It has been determined that bazlama made from contaminated flours with OTA levels of 5 and 10 μg/kg contained OTA levels exceeding 3 μg/kg when long‐term fermentation was not used. This is the maximum permitted limit set by the Turkish Food Codex and the European Commission, indicating that it is not suitable for consumption in this position.

## Graphical abstract

Wheat flour, intentionally contaminated with OTA at 5 and 10 μg/kg, was used to make sourdough flatbread (SFB). Using the HPLC‐FLD method, the study observed the effects of different fermentation times, baking, and storage at room temperature for 0, 5, and 10 days on the levels of OTA in the SFB. It was concluded that different storage times did not have a significant effect on OTA and that prolonged fermentation (at least 3 h) was more effective, especially in flours with high OTA contamination.

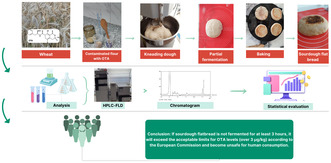

## 1 INTRODUCTION

Cereal crops, like wheat, and various foodstuffs can contain microorganisms as part of their natural structure, or they can become contaminated from external sources (Çelik, 2008). Ochratoxin A (OTA) is the most common mycotoxin formed when cereals are contaminated by various fungi of the genera *Aspergillus* spp. and *Penicillium* spp. (European Commision, 2022). OTA formation is not limited to raw materials but also occurs in processed products due to the thermostability of mycotoxin (Duarte et al., 2010). Cereal‐based processed products provide a significant portion of the daily calorie needs of many people globally and are used as a staple food (Khaneghah et al., 2022). Among these cereal products, bread is one of the most important and has many varieties (Duarte et al., 2010). There are many types of flat breads in the world known by different names, such as tandoori, pita, tortilla, lavash, bazlama, and yufka. The traditional flat breads of Turkey are pita bread and bazlama (traditional Turkish flat bread). Bazlama is a yeasted, single‐layered, cream‐yellow‐colored flat bread with an average diameter of 10–20 cm (Emeksizoğlu, 2016). While bazlama is mostly produced and consumed in villages, it has started to be consumed frequently in cities today. The quality of the flour used in the production of bazlama is the most important factor in determining the quality of the final product.

Ochratoxin A‐producing molds can contaminate food during the harvesting of cereal products and/or during post‐harvest storage (Girgin et al., 2001). Although it is impossible to completely eliminate all causes of mold infection, it is possible to reduce or even prevent the conditions that lead to mold growth. Drying grains to below 15% moisture level (about 0.8% water activity) and maintaining this moisture level throughout storage is one of the most fundamental protection methods. Delays in drying can put the grain at risk and cause problems during and/or after storage (Duarte et al., 2010). Physical treatments such as cleaning, peeling, decontamination, washing, and heat treatment, as well as the use of natural and synthetic chemicals, as well as biological methods including microorganisms such as *Bacillus* spp., lactic acid bacteria, and propionic acid bacteria, are used to reduce risks and/or stop toxin growth. (Abedi et al., 2022; Sabuncuoğlu et al., 2008; Tosun, 2015). Biological method(s) have been recommended for decontamination due to their various advantages that are environmentally friendly, efficient, cost‐beneficial, and maintain nutritional quality (Abedi et al., 2022).

The fact that OTA is mostly found in cereals and flours produced from contaminated cereals increases the possibility of its presence in bread types such as bazlama made from these flours. OTA is also transmitted to humans through the consumption of these products. According to the European Commission (2002) and the Turkish Food Codex (2011), the maximum acceptable limit of OTA is 5 g/kg in unprocessed raw cereals and 3 g/kg in all products made from unprocessed grain (including cereals intended for direct human consumption and processed cereal products). However, some studies found that the OTA levels in flour and bread (Cengiz et al., 2007; Koç, 2016) and commercial corn and wheat products (Majeed et al., 2018) were higher than these maximum values. Consequently, the presence of mycotoxin in raw materials is a concern within the food industry as it causes economic losses and public health problems. Food processing plays a critical role in reducing mycotoxin contamination of raw materials and food safety (Khaneghah et al., 2022). For this reason, although the main objective is to produce flours with low or no mycotoxin content, it is also important to determine the rate of toxin reduction during production.

To the best of our knowledge, no study has been conducted to investigate the effects of the different fermentation times, baking conditions, and storage conditions on the presence of OTA in Turkish traditional flat bread called “bazlama,” produced with sourdough. This study aimed to investigate the effect of production stages, including fermentation time (at 25 ± 5°C for 0, 1.5, and 3 h), baking on a sheet at 300 ± 5°C for a shorter duration (10 min), and storage (at room conditions of 23–25°C for up to 10 days), on the decrease in OTA concentrations in sourdough bazlama made from flour contaminated with OTA at varying levels.

## 2 MATERIALS AND METHODS

### 2.1 Materials

Bread wheat flour without additives obtained from Hediye Un Industry Co. (Isparta) was taken from freshly milled flour and kept at 25°C for 3 weeks for ripening. Refined crystalline salt was purchased from the local market in accordance with the TS 933 (TSE, 2003) Edible Salt Standard. OTA Standard solution (Sigma‐Aldrich) was supplied in 10 μg/mL liquid form and stored at −18°C under refrigerated conditions until used for analysis. The ImmunoClean C OTA column (Aokin) containing specific antibodies against OTA was used as the OTA immunoaffinity column. Methanol and glacial acetic acid (Merck), acetonitrile (Honevwell), and phosphate buffered saline (PBS, Sigma) were purchased to be of analytical purity.

### 2.2 Production method of sourdough

Sourdough used in the production of bazlama was produced in a 5‐day process by making some modifications to the method given by Özuğur (2011). On the first day, bread wheat flour, water, and chickpeas (100:150:3, w/v/w) were mixed and left for fermentation in an incubator (Nüve) at 30°C for 2 days. On the third and fourth days, flour and water (100:100, w/v) were added and mixed, and fermentation was continued under the same conditions. It was determined that the pH value of the sourdough prepared on the fifth day decreased to 3.9 ± 1. After reaching this pH value, the sourdough was ready to be used in the production of bazlama.

### 2.3 Preparation of an OTA intermediate stock solution

Intermediate stock solutions of 5 and 10 μg/kg concentrations were prepared from the OTA main stock standard solution at a concentration of 10 μg/mL to contaminate the flour sample and to be used for calibration in HPLC. The prepared intermediate stock solutions were kept at +4°C under refrigerated conditions and allowed to reach room temperature before use.

### 2.4 Contamination of wheat flour with OTA

Bread wheat flour without additives was tested for cleanliness (below the detection limit) in terms of toxin by OTA analysis, and it was found to be clean. The flour was then contaminated by adding 5 and 10 μg/kg of OTA intermediate stock solution, and a mask, gloves, and laboratory goggles were used for safety. After adding OTA, the contaminated flour was homogenized well, and bazlama dough was prepared from this flour. During the bazlama production process, samples were taken from the kneaded dough, fermented dough, and baked bazlama, and the presence of OTA was analyzed. The amounts of 5 and 10 μg/kg OTA in flour were analyzed at the end of kneading, fermentation, cooking, and storage. Analyses were carried out in three replicates with two parallels, and a total of 108 bazlama breads were analyzed.

### 2.5 Bazlama production method

**FIGURE 1:**
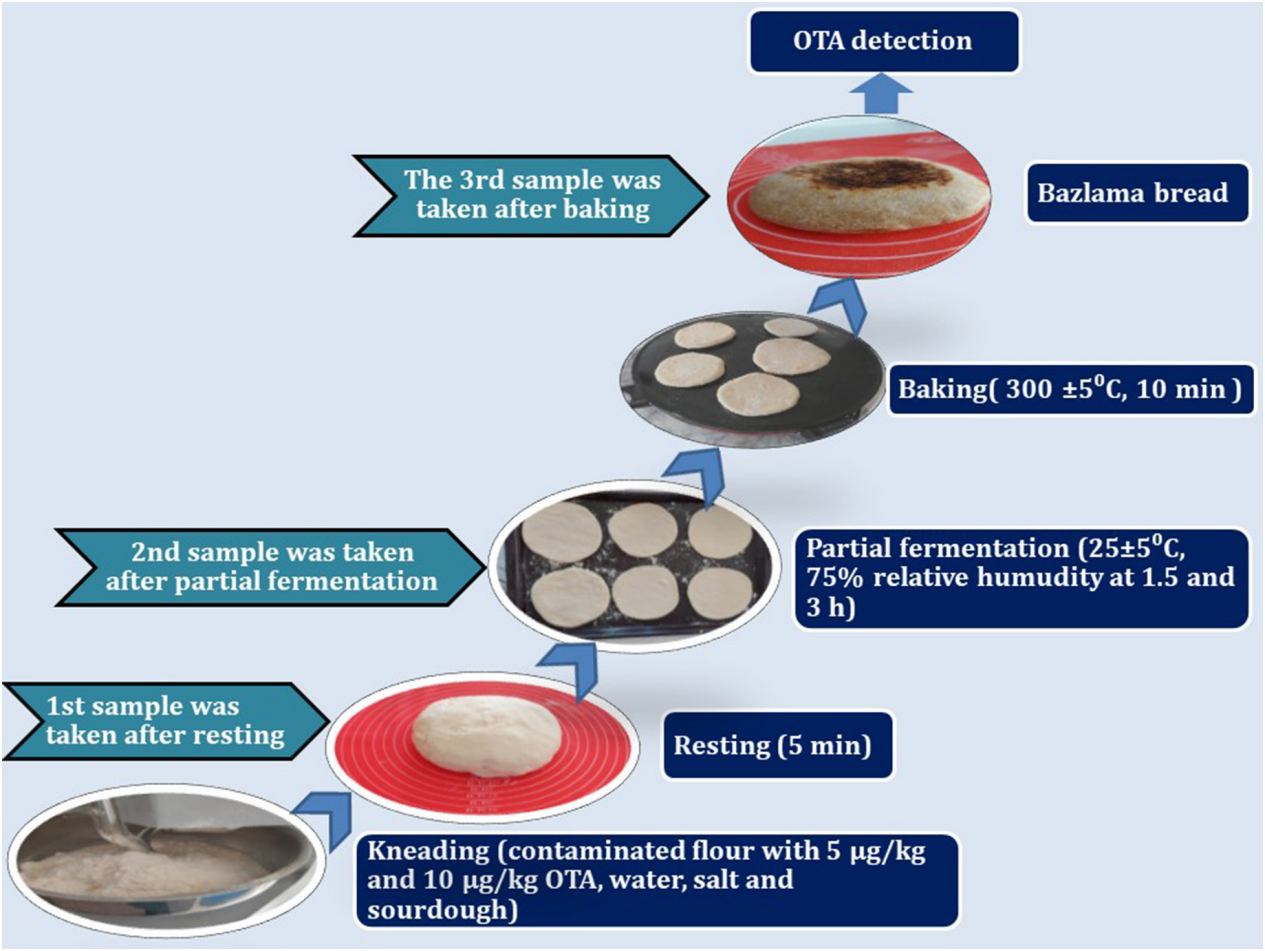
Bazlama making stages and sampling plan.

Bazlama dough was made with wheat flour (contaminated with OTA at 5 and 10 μg/kg), salt, sourdough, and water (50:1.5:32.5:0.75, w/w/w/v). The amount of water to be added was determined by a farinograph (Brabender). A diosna‐type dough kneading machine (Günsa) with a spiral shaft was used for the kneading process. The yeast was placed in the kneader with approximately 20% of the water and allowed to dissolve. The flour was added and kneaded at slow speed for 5 min, then the salt was dissolved with the remaining water and kneaded at fast speed for 5 min. It was then left to undergo mass fermentation for 5 min. After kneading, OTA analysis was performed on samples taken from the dough (dough‐0) before being left for fermentation. Doughs were divided into equal pieces of 150 g, and then these doughs were round‐shaped, 140 mm in diameter, and 7 mm thick. After shaping, bazlama doughs were left for fermentation at 25 ± 5°C for 1.5 h (dough‐1) and 3 h (dough‐2) by covering them with a damp cloth. Two different fermentation times, 1.5 and 3 h, were applied, and the effect of the fermentation process on OTA reduction was investigated. After fermentation, bazlama doughs were baked on an electric baking sheet (Orhome) at a temperature and time (300 ± 5°C for 10 min) determined by preliminary trials. The baked bazlama samples (bazlama‐1, bazlama‐2) were cooled at room temperature and then placed in double‐layer polyethylene packages and analyzed for OTA. Bazlama production stages and sampling plan are shown in Figure 1.

### 2.6 Storage of bazlama samples

Bazlama samples produced from 5 to 10 μg/kg OTA‐contaminated flour and at two different fermentation times (1.5 and 3 h) were stored at 25 ± 2°C for 0, 5, and 10 days, and the effect of this storage process on the presence of OTA was investigated.

### 2.7 Determination of the presence of OTA in bazlama samples

In this study, AOAC Method 2000.03 (AOAC, 2000) was applied with some modifications as a result of literature reviews (Chung & Kwong, 2007; Entwisle et al., 2000; Juan et al., 2007, 2008; Ng et al., 2009; Savaş, 2016) for the determination of OTA in flour, dough, and bazlama samples. Extraction was performed according to the instructions set out in the immunoaffinity column usage procedure (Aokin Rapid Analysis Systems, 2022).

A precision balance (And GR 200) was used to weigh 25 g of homogenized dough or bazlama samples. Then, 100 mL of the acetonitrile/water (60/40) mixture was added and transferred to a blender (Waring Commercial Laboratory Blender). The mixture was mixed at a high speed of 22,000 rpm for 2 min. It was then filtered through Whatman 113 (Cytiva) filter paper. After filtration, 4 mL of the filtrate was placed in a 50‐mL centrifuge tube with 44 mL of PBS (Sigma) and shaken for 5 s. The Immuno Affinity Column was placed on a pump stand, and a 50‐mL syringe was attached to the top cap. The extract, diluted with PBS buffer, was transferred to an ImmunoClean C OTA column and passed through the column at 3 mL/min. After the entire extract was passed through the Immuno Affinity Column, 10 mL of ultrapure water was washed with 1–2 drops per second to remove impurities from the sample. 1 mL of methanol was added to the column and incubated for 3 min by slowing down the flow to stop, then 3 mL of methanol (1 mL three times) was passed at 1 drop per second. Elouate was evaporated under nitrogen gas at 40°C to complete dryness. The resulting residue was dissolved in 1 mL of mobile excess and mixed with vortex (Nüve) for 1 min. After mixing, the eluate in the vial was injected into the HPLC device with a fluorescence detector in the amount of 100 μL.

### 2.8 OTA determination by HPLC

The extracts obtained from the extracted dough and bazlama samples were analyzed by an HPLC device using a Shimadzu (Japan) brand HPLC device in the Isparta Food Control Laboratory Directorate Mycotoxin and Residue Laboratory. The device includes an RF‐10AXL liquid fluorescence detector, a DGU‐20A3 degasser, an LC‐20 AD LC pump, a SIL‐20A HT auto sampler, and a CTO‐10AS VP column oven. The operating conditions of the HPLC device are as follows: Flow rate: 0.9 mL/min; injection volume: 100 μL; column temperature: 45°C; wavelength: FLD EX: 333, EM: 477 nm (Emission‐Emitted wavelength); Column: 250 × 4.6 mm, 5 μ, C18 (ACE ACE‐121‐2546 or equivalent); mobile phase: acetonitrile–water–acetic acid (48/51/1, v/v/v); analysis time: 15 min; and pressure: minimum 0 bar, maximum 200 bar. The calibration curve is based on the analyses of six standard solutions made from appropriate volumes of the reference solution; the OTA standard covers the range of 0.5–20 μg/kg. The peak areas corresponding to these concentrations injected into the HPLC device formed a calibration curve at six different points with the Shimadzu LCsolution Calibration Curve software in the device. The correlation coefficient (*r* ^2^) value was found to be .99997. This calibration curve was used to analyze OTA in flour, dough, dough fermented at different times, and bazlama samples. The signal‐to‐noise ratio was used to calculate the limit of detection (LOD) and limit of measurement (LOQ). The LOD was used as three times the signal‐to‐noise ratio, and the LOQ as 10 times the signal‐to‐noise ratio. As a result of the calculations, the LOD was determined to be 0.55 μg/kg and the LOQ to be 1.83 μg/kg.

### 2.9 Recovery

**FIGURE 2:**
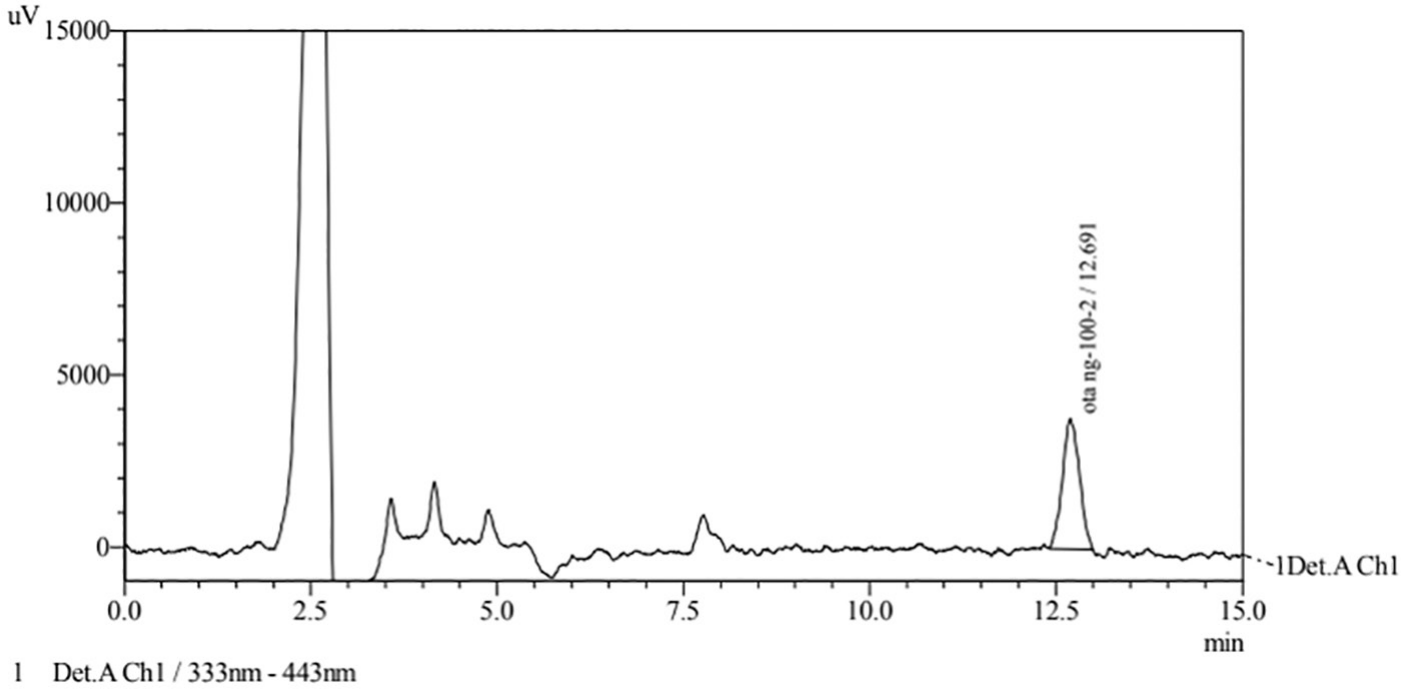
Recovery chromatogram image for 10 μg/kg.

Flour samples taken from Hediye Flour Factory and analyzed for the presence of OTA were used for recovery studies. For the recovery data, according to the performance criteria for OTA in the Turkish Food Codex Communiqué on Sampling, Sample Preparation, and Analysis Method Criteria for the Official Control of Mycotoxin Levels in Foods No. 2018/10 (Turkish Food Codex, 2018) and the European Commission (2006), recovery rates for OTA concentrations ≥1 should be between 70% and 110% and recovery rates for <1 should be between 50% and 120%. The samples' average recovery rates at 5 and 10 μg/kg concentrations (for OTA concentrations ≥1) were 78.4% and 92.85%, respectively. These recovery rates were in line with what the Turkish Food Codex and the European Commission say are acceptable levels of performance. The typical chromatogram image obtained is shown in Figure 2.

### 2.10 Statistical evaluation

The “Shapiro–Wilk normality test” was used to determine whether the data were normally distributed. An independent sample *t*‐test and a repeated‐measures ANOVA test were used in the statistical evaluation of the normally distributed data, and the TUKEYHSD test was used to determine the difference between the experimental groups. All statistical evaluations were carried out in the Rstudio (v2022.07.1) computer program (RStudio Team, 2022) at the 0.05 significance level.

## 3 RESULTS AND DISCUSSION

The changes in OTA at the subsequent stages of the bazlama production process, after kneading (dough‐0), fermentation for 1.5 and 3 h (dough‐1, dough‐2) and baking (bazlama‐1, bazlama‐2) are given in Table 1.

**TABLE 1:** The mean concentrations of ochratoxin A (OTA) determined in the doughs and baked bazlama samples.

Sample name	OTA concentration mean ± SE	OTA concentration mean ± SE	*p* *
Flour (starter)	5 μg/kg	10 μg/kg	
Dough‐0	3.69 ± 0.098^a^	6.44 ± 0.176^a^	.003
Dough‐1	3.53 ± 0.114^ab^	5.78 ± 0.228^ab^	.016
Dough‐2	3.36 ± 0.177^ab^	5.25 ± 0.189^bc^	.009
Bazlama‐1	3.11 ± 0.090^ab^	5.37 ± 0.202^bc^	.014
Bazlama‐2	2.96 ± 0.138^b^	4.59 ± 0.209^c^	.023

*Note*: Dough‐0, dough sample taken immediately after kneading and not left for fermentation; Dough‐1, Sample taken from dough left for fermentation for 1.5 h; Dough‐2, Sample taken from dough left for fermentation for 3 h; Bazlama‐1, Bazlama sample baked after 1.5 h of fermentation; Bazlama‐2, An example of bazlama baked after 3 h of fermentation; A statistical difference was determined between the averages marked with different letters in the same column (*p* < .05).

* Result of the *t*‐test for the comparison of OTA levels of dough groups with 5 and 10 μg/kg initial OTA additions after they were transformed into the same product.

According to Table 1, when two different concentrations (5 and 10 μg/kg) were analyzed separately, the difference was found to be significant (*p* < .05) according to the *t*‐test result for the comparison of the OTA levels of the initial OTA‐added dough groups after they turned into the same product. This difference, which is determined statistically, is related to the initial concentration of OTA at the beginning. At the end of the fermentation process, this ratio decreases in proportion to the OTA concentration. In light of these findings, it is possible to say that the amount of OTA detected decreased as the fermentation time increased in bazlama doughs made from flour contaminated with OTA. It is noteworthy that the decrease in OTA varies according to the amount of toxin. Abedi et al. (2022) investigated the binding capacity and stability of OTA (10 μg/kg) with lactic acid bacteria and found that binding was dependent on many factors, such as strain, toxin amount, cell hydrophobicity, pH value, and bacterial viability.

### 3.1 Effect of fermentation on the OTA level

An important difference was found between the averages marked with different letters (a–c) in the same column during the bazlama production process for the two different amounts of OTA (5 and 10 μg/kg).

The amount of OTA was determined as 3.69 μg/kg in the dough sample (dough‐0) taken from the dough mass kneaded after the flour was contaminated with 5 μg/kg OTA (without any fermentation, i.e., as soon as the kneading was finished), and 3.53 and 3.36 μg/kg in the dough samples (dough‐1 and dough‐2) taken after this dough was left to ferment for 1.5 and 3 h, respectively. No statistically significant difference (*p* > .05) was found between the samples after fermentation of the kneaded dough for 1.5 h (dough‐0 to dough‐1) and after fermentation for 3 h (dough‐0 to dough‐2). As a result, it was found that if the flour used in the production of bazlama is contaminated with 5 μg/kg (low concentration) OTA, short (1.5 h) or long (3 h) fermentation times will not have any effect on reducing this contamination. As a result of the measurements carried out at the end of contamination of flour with 10 μg/kg OTA, the amount of OTA was determined to be 6.44 μg/kg in the dough sample that was not subjected to any fermentation process (dough‐0), 5.78 μg/kg (dough‐1) and 5.25 μg/kg (dough‐2) at the end of 1.5 and 3 h of dough fermentation, respectively. As can be seen in Table 1, there was no statistically significant difference between 0 and 1.5 fermentation times, while a significant difference was found between 0 and 3 h of fermentation time (*p* < .05).

The percent reduction of OTA concentration after kneading of bazlama doughs was analyzed, and it was determined that the OTA concentration decreased by 26.2% and 35.56%, respectively, when the flour was contaminated with 5 and 10 μg/kg OTA. When the doughs produced from flour contaminated with 5 μg/kg OTA were fermented for 1.5 and 3 h, their OTA concentration decreased by 4.17% and 8.88%, respectively. After 1.5 h of fermentation of doughs contaminated with 10 μg/kg OTA (dough‐0, dough‐1), OTA concentration decreased by 10.24% and after 3 h of fermentation (dough‐0, dough‐2) by 18.4%. As in the case of 5 μg/kg, a decrease of approximately two times was determined, and a statistically significant difference was found between dough‐0 and dough‐2 (*p* < .05). It is found that if the flour used in the production of bazlama is contaminated with high concentrations of OTA (10 μg/kg), a longer fermentation time (at least 3 h) will have an effect on reducing the OTA concentration.

Valle‐Algarra et al. (2009) reported that 60 min of fermentation at 30°C in bread doughs made by using *Saccharomyces cerevisiae* decreased the average amount of OTA by 31.1%. On the other hand, Savaş (2016) reported that 75 min of fermentation at 30°C decreased the average amount of OTA by 43.7%, and this change in OTA level was significant. Cecchini et al. (2006) reported that the level of OTA in the medium decreased significantly during the alcohol fermentation of wine. Milani and Heidari (2017) investigated the effect of fermentation with different yeasts on the stability of ochratoxin during bread‐making. The percentage of OTA reduction after fermentation with compressed yeast, instant dry yeast, active dry yeast, and sourdough for 90 min at 30°C was found to be 45.83%, 16.66%, 8.69%, and 9.09%, respectively. While instant dry yeast, active dry yeast, and sourdough were not effective in OTA reduction, compressed yeast caused significant OTA reduction in fermentation. The effect of fermentation on OTA degradation was due to a drop in pH and the presence of CO2‐producing yeast species that could make metabolites after fermentation. Vidal et al. (2014) investigated the change in OTA with the use of sourdough in bread making, and they found a slight decrease (29%) in OTA concentration during the fermentation of dough. Scudamore et al. (2003) and Vidal et al. (2014) observed a high stability of OTA during dough fermentation. Nevertheless, the results of Scudamore et al. (2003) and Vidal et al. (2014) correlate well with ours, as 1.5 and 3 h fermentation had no effect on OTA at low concentrations. The decrease in OTA concentration during fermentation has been found to depend on the strain involved in the fermentation caused by lactic acid bacteria (Shetty & Jespersen, 2006) and yeasts (Cecchini et al., 2006; Westby et al., 1997), but Mozaffary et al. (2019) report that it depends on the amount of yeast and fermentation temperature.

Studies on binding chemistry have shown that the polysaccharide moiety on the cell wall surface plays an important role in the binding of toxins to the surface in both *S. cerevisiae* and lactic acid bacteria. However, in general, fundamental differences in their structures have shown that they exhibit different abilities in terms of toxin binding capacity and strength (Shetty & Jespersen, 2006). In our study, while the effect of 1.5 and 3 h fermentation was not observed in the bazlama made with flour at a concentration of 5 μg/kg, the effect of 3 h fermentation was observed in the bazlama made with flour at a concentration of 10 μg/kg. Based on these results, we can say that the amount of OTA found decreased as the fermentation time increased in bazlama doughs made from flour that was contaminated with OTA. It is important to note that the decrease in OTA depends on the amount of toxin.

### 3.2 Effect of baking on OTA levels of bazlama

Ochratoxin A levels of bazlama samples produced from flour contaminated with 5 μg/kg OTA were determined after baking as 3.11 μg/kg for bazlama‐1 (fermented for 1.5 h) and 2.96 μg/kg for bazlama‐2 (fermented for 3 h). On the other hand, these values were found to be 5.37 μg/kg for Bazlama‐1 and 4.59 μg/kg for Bazlama‐2, which were produced with flour contamination with 10 μg/kg OTA (Table 1). When the effect of baking at 300 ± 5°C for 10 min on OTA concentration (from dough‐1 to Bazlama‐1; from dough‐2 to Bazlama‐2) was analyzed for both 5 and 10 μg/kg, no statistical difference was found (*p* < .05). The addition of 5 μg/kg of flour‐contaminated doughs, followed by a fermentation period of 1.5 h, resulted in an 11.96% reduction in the OTA concentration of bazlama samples. Similarly, a fermentation period of 3 h resulted in an 11.89% reduction in the OTA concentration. Ten μg/kg flour‐contaminated doughs baked after fermentation for 1.5 h decreased the OTA concentration of bazlama samples by 7.05%, and 3 h of fermentation further decreased it by 12.63%. It is known that OTA is stable against heat due to its stable structure, and its melting point is 169°C. In this study, baking of bazlama samples at 300 ± 5°C for 10 min was not statistically effective on the OTA level. Ten minutes for making was not sufficient to reduce OTA. Due to the high thermostability of OTA, it was determined that it did not cause a significant increase or decrease in OTA content during baking.

It has been reported that thermal treatment of OTA‐contaminated wheat does not completely eliminate the toxin but reduces the OTA concentration by >50% (Boudra et al., 1995). Valle‐Algarra et al. (2009) subjected dough obtained from flour samples contaminated at 2, 5, and 10 μg/kg to baking at different temperature/time combinations (190°C/50 min, 207°C/40 min, 223°C/35 min, and 240°C/30 min) after fermentation. The researchers reported that there was no significant difference in the reduction in toxin concentration between different temperature and time treatments, and the average reduction value was 32.9%. Mozaffary et al. (2019) found the OTA loss during baking to be 19.21% during bread making. Bryła et al. (2021) determined the decrease in OTA concentration during pizza dough production as 13.5% for pizza dough baked at 320°C and 21.1% for pizza dough baked at 370°C. In some studies, mycotoxin was found to be stable at high temperatures and did not decrease during heating (Milani & Heidari, 2017; Osborne et al., 1996; Scudamore et al., 2003; Vidal et al., 2014), while in others, an increase in ochratoxin concentration was found (Khaneghah et al., 2022; Peng et al., 2015; Yu et al., 2020). Khaneghah et al. (2022) reported in their study that there was a significant increase in the level of OTA from cracker dough to the baking stage and that the food processing stage causes toxins to interact with the food matrix and release latent mycotoxins. The significant increase in the mean OTA level during the baking process (from fermented dough to crackers) was explained to be due to the release of latent OTA in flour, degradation, or alteration of the OTA matrix.

The fact that different results were found in the studies makes it difficult to compare our findings. Because different parameters such as formulation, shape, size, and height of the dough, aeration, type and amount of microorganisms in fermentation, baking temperatures and time, etc. lead to different results, Çelik (2008) reports that dihydroisocoumarin and phenylalanine compounds in the chemical structure of ochratoxins are linked to each other by amide bonds, and this causes them to gain a very stable structure against heat and hydrolysis.

Tosun (2015) explains that OTA maintains its steady state in food processing stages such as boiling, fermentation, cooking, frying, and the application of heat. The fact that cooking had no effect on OTA concentration in our study is similar to the observations of Milani and Heidari (2017), Osborne et al. (1996), Scudamore et al. (2003), and Valle‐Algarra et al. (2009).

### 3.3 Effect of storage on OTA levels of bazlama

The effect of 25 ± 5°C storage for 0, 5, and 10 days on the OTA level in bazlama samples is given in Table 2.

**TABLE 2:** Effect of storage on ochratoxin A (OTA) levels of bazlama samples.

Fermentation time (h)	Storage time (days)	OTA concentration		* *p* Values
		5 μg/kg	10 μg/kg	
		Ort ± SE	Ort ± SE	
1.5	0	3.11 ± 0.09	5.37 ± 0.20	.008
	5	2.86 ± 0.12	5.13 ± 0.17	.002
	10	2.74 ± 0.10	4.93 ± 0.16	.003
3	0	2.96 ± 0.13	4.59 ± 0.20	.014
	5	2.75 ± 0.11	4.30 ± 0.16	.007
	10	2.67 ± 0.09	3.96 ± 0.08	.002

*Note*: Statistical difference was determined between the averages marked with different letters in the same column (*p* < .05).

* Result of *t*‐test for the comparison of the mean OTA levels at the end of the same storage period of the products with initial OTA level of 5 and 10 μg/kg subjected to the same fermentation period.

There was no statistical difference (*p* > .05) between the mean OTA levels measured at the end of the storage periods (0, 5, and 10 days) of the Bazlama samples obtained after 1.5 and 3 h of fermentation of the products to which 5 and 10 μg/kg OTA were initially added (Table 2). In this case, it was determined that storing the bazlama under room conditions for up to 10 days had no statistical effect on the OTA concentration of both 5 and 10 μg/kg. When two different concentrations (5 and 10 μg/kg) are analyzed separately in Table 2, the concentration ratios of the OTA analysis results of bazlama subjected to 1.5 and 3 h of fermentation and baked showed statistical difference between the process steps according to *t* test in 0, 5, and 10 days storage period (*p* < .05). This statistically determined difference is related to the OTA concentration in the flour at the beginning, and it is due to the fact that the low OTA concentration decreases to lower levels during the storage of the bazlama, while the high OTA concentration remains at higher levels. At 1.5 h of fermentation time, the joint relationship (interaction) of storage day and fermentation time to the end of experiment OTA levels was found to be statistically significant (*p* < .05) between day 0 and day 10, but insignificant (*p* > .05) in other comparisons. At 3 h of fermentation time, the joint relationship (interaction) of storage day and fermentation time to the end of trial OTA levels was found to be statistically significant (*p* < .05) between day 0 and day 10, but insignificant (*p* > .05) in other comparisons.

Abedi et al. (2022) stated that non‐living lactic acid bacteria strains could detoxify OTA in food, and Piotrowska (2014) found that non‐living (inactivated) lactic acid bacteria reduced OTA in the environment by 46.2–59.8%. In this study, the slight decrease in the OTA level of the bazlama obtained after two different fermentations containing different concentrations of toxin indicates that the dead lactic acid bacteria continue to reduce the OTA in the environment, but mycotoxin still maintains its stability. In addition, Berthiller et al. (2013) report that mycotoxins change their structure through biological and chemical reactions that occur during food processing (such as fermentation or cooking) and become undetectable by conventional analytical methods. This causes them to appear to cause a decrease in OTA levels.

## 4 CONCLUSION AND FUTURE TRENDS

In this study, although a general decrease in OTA level was found, fermentation and baking did not have a significant effect. Only fermentation at a high concentration (10 μg/kg) for 3 h (between dough and dough 2) was found to have an effect. The decrease in OTA level indicates that OTA is slightly reduced in the applied processes but cannot be completely removed from the environment. Processes such as baking or fermentation can disrupt the structure of OTA, perhaps causing it to transform into more toxic compounds (metabolites). This reduction only shows that the analysis method used to analyze the product was unable to detect these compounds (or metabolites), and this aspect is still subject to further investigation. In addition, since the type of lactic acid bacteria used in fermentation using sourdough affects OTA binding, further research is required to determine the type of yeast that will be most effective during fermentation. Furthermore, by extending the fermentation period by up to 24 h, more research on the variation in bazlama's OTA levels can be conducted.

It was determined that bazlama produced from flours to which OTA was added at both 5 and 10 μg/kg concentrations (excluding 5 μg/kg during 3 h of fermentation) during the production of bazlama contained OTA above 3 μg/kg, which is the maximum acceptable limit by TGK and the European Commission. Although the OTA concentration decreased between 45% and 55% during the production process of bazlama, it was determined that it remained above the legal limits and that it was not suitable for human health. With this result, necessary studies should be carried out to prevent the formation of OTA before grinding wheat into flour. Contamination of foodstuffs and animal feeds with OTA is a serious problem not only in Turkey but all over the world. Foods with high mycotoxin levels have special importance in terms of the health risks they pose. OTA levels vary climatically and regionally, but they also vary according to the production process of the products obtained from them. In order to prevent OTA formation in foods, mold contamination should be prevented or minimized during and after harvest. When necessary precautions are taken to prevent the formation of OTA and detoxification is provided by physical, chemical, and biological techniques, toxin levels will be significantly reduced. This will benefit both the country's health and its economy. The aim is to try to minimize all mycotoxin‐related risks.

To be able to produce healthy products and achieve quality in food production, it is necessary to follow an infection control strategy based on HACCP (Hazard Analysis Critical Control Point) and Barrier Technology Systems. These systems must be spread from pre‐harvest to post‐harvest. In addition, the absence of OTAs in exported products and the associated low RASSF notifications are fundamental necessities to ensure today's global trade, economy, and, more importantly, consumer safety requirements.

## AUTHOR CONTRIBUTIONS

**Nazlı Özdemir:** Conceptualization (equal); data curation (equal); investigation (equal); methodology (equal); visualization (equal); writing – original draft (equal). **Hülya Gül:** Conceptualization (equal); data curation (equal); investigation (equal); methodology (equal); project administration (lead); supervision (lead); visualization (equal); writing – original draft (equal); writing – review and editing (lead).

## FUNDING INFORMATION

This work was supported by the Süleyman Demirel University Scientific Research Projects Coordination Unit (Project Number FYL‐2021‐8457).

## CONFLICT OF INTEREST STATEMENT

None.

## Data Availability

The corresponding author will share the data upon request.
